# Synthesis, Characterization, and Antifungal Activity of Silver Nanoparticles Embedded in Pullulan Matrices

**DOI:** 10.3390/ma14227041

**Published:** 2021-11-20

**Authors:** Olga Burduniuc, Andra-Cristina Bostanaru, Mihai Mares, Gabriela Biliuta, Sergiu Coseri

**Affiliations:** 1 Department of Microbiology, Virusology and Immunology, “Nicolae Testemitanu” State University of Medicine and Pharmacy, 165, Stefan cel Mare blvd., MD 2001 Chisinau, Moldova; 2 National Public Health Agency, 67A Gheorghe Asachi, MD 2028 Chisinau, Moldova; 3 Laboratory of Antimicrobial Chemotherapy, Faculty of Veterinary Medicine, “Ion Ionescu de la Brad” University of Life Sciences (IULS), 8 Mihail Sadoveanu Alley, 700489 Iasi, Romania; acbostanaru@uaiasi.ro; 4 “Petru Poni” Institute of Macromolecular Chemistry, 41A Grigore Ghica Voda Alley, 700487 Iasi, Romania

**Keywords:** antifungal activity, silver nanoparticles, pullulan, oxidation

## Abstract

Steady developments made in nanotechnology-based products have facilitated new perspectives for combating drug-resistant fungi. Silver nanoparticles represent one of the most attractive nanomaterials in biomedicine due to their exclusive optical, electromagnetic, and catalytic properties and antifungal potency compared with other metal nanoparticles. Most studies show that the physicochemical parameters affecting the antifungal potential of AgNPs include the shape, size, surface charge, and concentration and colloidal state. For the present study, pullulan (P) and its oxidized counterpart (PO) have been selected as matrices for the silver nanoparticles’ generation and stabilization (AgNPs). The TEMPO (2,2,6,6-tetramethylpiperidin-1-yl radical)–sodium hypochlorite–sodium bromide system was used for the C6 selective oxidation of pullulan in order to introduce negatively charged carboxylic groups in its structure. The structure and morphology of the synthesized AgNPs were analyzed using FTIR and EDX. The main objective of this study was to elucidate the antifungal activity of AgNPs on the clinical yeasts isolates and compare the performance of AgNPs with the conventional antifungals. In this study, different concentrations of AgNPs were tested to examine antifungal activity on various clinical isolates.

## 1. Introduction

Fungal diseases have been underappreciated over the last few decades, which resulted in a significant impact on human health. Thus, pathogenic fungi infect billions of people worldwide, with deaths in excess of 1.5 million per year [1,2]. Disease burden ranges in severity from mild, superficial infections (e.g., skin, nail, and hair infections) to severe invasive fungal infections, which are disseminated infections involving different organs (e.g., brain, heart, and lungs) [3]. Most of the pathogenic fungi are opportunistic and cause disease under immunocompromised conditions such as long-term antibiotics administration, organ transplantation, cancer, treatments involving steroids, or HIV infections [4,5,6,7,8,9]. Despite some advances in treatment, invasive fungal infections persist as a common cause of morbidity and mortality in immunocompromised patients, especially affecting high-risk patients [10].

Silver and its related compounds were used for antibacterial and therapeutic applications for thousands of years [11,12,13]. Nanotechnology has recently become one of the most inspiring branches of science, which intersects with biology and medicine [14] and produces novel, functional materials such as nano-sized particles. Silver nanoparticles (AgNPs) have attained a primary role with the most appealing nanomaterials in biomedicine due to their large range of activities and their unique physical and chemical properties [15,16]. There is a considerable amount of literature that demonstrates their unique physical and chemical properties, and intensive studies have been conducted to investigate AgNPs properties and potential implementations as efficient antifungal agents [17]. In the last decade, most studies were focused on the synthesis of AgNPs with controlled size and shape, and a diversity of specific synthetic protocols have been proposed, including chemical, physical, or biological methods [18].

Initially, the research focused on synthesizing and characterizing AgNPs following chemical approaches, but nowadays, extensive studies concentrate on the biological effects and applications of AgNPs [19,20]. Therefore, recent studies demonstrate that the antimicrobial effect of AgNPs relies on physicochemical characteristics like size, shape, distribution, and concentration [21,22,23,24]. Regarding the silver nanoparticles molecular mechanism, some recent studies have demonstrated that Ag^+^ ions released into the cytoplasmic cavity of fungal cells disrupt the respiratory system with a significant impact on DNA replication mechanisms and the expression of genes involved in such processes [25,26,27]. AgNPs have unique properties in terms of toxicity, surface plasmon resonance, and electrical resistance [20]. Some studies note that one of the researchers’ concerns is the presumption of cytotoxicity of AgNPs to humans. However, most studies have shown that at low concentrations, silver nanoparticles exhibit no cytotoxicity to human cells; the toxicity may occur when increased doses are used [28,29,30].

In the last few years, increased interest was observed in the fabrication of AgNPs with potent antimicrobial activity using polysaccharides like chitosan [31], starch [32], agar [33], dextran [34], and guar gum [35]. These polysaccharides have the fundamental problem of low solubility and high viscosity and, hence, cannot be employed in the large-scale production of silver nanoparticles. On the other hand, polysaccharides with many hydroxyl groups have been the potential candidate as a reducing and stabilizing agent for AgNPs [36]. Thereby, pullulan proved to be an excellent candidate for this purpose. Pullulan is a linear homopolysaccharide synthesized extracellularly by the polymorphic fungus, *Aureobasidium pullulans*, which is highly water-soluble, with promising utilization in the food and pharmaceutical sectors. Pullulan’s spectacular adhesive and film forming abilities are successfully employed for fabric compression moldings, fibers, drug delivery systems, and edible films [37,38]. Green synthesis of AgNPs reduced and stabilized by pullulan was rarely reported. In the present study, pullulan and oxidized pullulan were tested for the syntheses of AgNPs, acting as both reducing and stabilizing agents. The synthesized AgNPs were characterized using Fourier transform infrared (FTIR) and energy dispersive X-ray spectroscopy (EDX). The scope of the study, in contrast with our previous work [36], was to test the AgNPs ability to act as antifungal agents towards 100 different clinical isolates belonging to 19 species and 5 genera. Our previously paper successfully demonstrates that pullulan (oxidized pullulan) silver nanoparticles have antibacterial activity against two types of bacteria: *S. aureus* and *E. coli* [36].

## 2. Materials and Methods

### 2.1. Materials 

Pullulan (TCI Europe, Mw = 150 kDa), 2,2,6,6- tetramethylpiperidin-1-yl (TEMPO), sodium hypochlorite (NaClO, 9% chlorite, Chemical Company, Iasi, Romania), sodium bromide (99% Alfa Aesar, Bucuresti, Romania), and silver nitrate (AgNO_3_) (≥99% Sigma-Aldrich, Bucuresti, Romania) were used without further purification. Fluconazole, RPMI-1640 medium, 3-(*N*-morpholino) propanesulfonic acid (MOPS), glucose, and dimethylsulfoxide (DMSO) were obtained from Sigma–Aldrich. All solutions were prepared using deionized water. 

### 2.2. Fungal Isolates

In this study, 100 different clinical isolates belonging to 19 species and 5 genera (namely Candida, Cryptococcus, Magnusiomyces, Saccharomyces, and Trichosporon) were used. Two genera were represented by a single species each*—Magnusiomyces capitatus* and *Trichosporon asahii.* Saccharomyces genus was represented by one single species—*S. cerevisiae* with 3 isolates. The most abundant genus in this study was *Candida,* with 15 species and 94 clinical isolates. Most isolates represented the species *C. glabrata*—27, followed by *C. albicans*—23, and *C. parapsilosis* with 16 isolates. *C. krusei* is represented by 5 isolates; *C. guilliermondii* and *C. tropicalis*—by 4 isolates each; *C. lusitaniae*—by 3 isolates; *C. famata*, *C. kefyr*, *C. norvegensis* and *C. utilis*—by 2 isolates each; and *Cryptococcus neoformans*, *C. pulcherrima*, *C. pelliculosa*, *C. dubliniensis*, *C. robusta*—by one isolate each, obtained from the Romanian Type Culture Collection. These isolates were stored in 20% glycerol at −80 °C.

### 2.3. Pullulan Oxidation (PO)

Pullulan oxidation was accomplished as follows: a water solution (50 mL) containing 0.5 g pullulan TEMPO (0.1 mM/g pullulan) and NaBr (1 mM/g pullulan) was prepared, under stirring at room temperature. Afterwards, a sodium hypochlorite solution (8%, 10 mM/g pullulan) was brought to pH ~10 (using 4 M aqueous HCl) and added to the aqueous mixture containing pullulan. The reaction is pH-dependent, so the pH value was maintained strictly at 10 using a 2 M sodium hydroxide solution. The reaction was stopped after 2 h, with 5 mL of ethanol. A large volume of acetone was used for the precipitation of the reaction product. The precipitate was collected by centrifugation, re-dissolved in water, and dialyzed using a polyethersulfone Millipore ultrafiltration membrane (cut-off: 12.500 g/cm). The diafiltration was stopped when the conductivity of the filtrate was lower than 10 µS m^−1^; the product was recovered by freeze-drying.

### 2.4. Synthesis of Silver Nanoparticles (AgNPs)

To prepare AgNPs, several concentrations of pullulan (1%, 5%, and 7%) and oxidized pullulan (1%) were mixed with an aqueous solution of silver nitrate (AgNO_3_) freshly prepared in deionized water under magnetic stirring (Laboratorium, Bucuresti, Romania) to achieve the final concentration of 5 mM (AgNPs/P-1%), 5 mM (AgNPs/PO-1%), 0.5 mM (AgNPs/P-5%), and 1.6 mM (AgNPs/P-7%). For AgNPs preparation, a 10 mL aqueous polymer solution was placed in a 50 mL three-neck flask (with protection against the light) then 10 mL of the fresh AgNO_3_ solution was added dropwise under vigorous stirring at 25 °C. After preparation, the solutions were dried at 60 °C.

### 2.5. Characterization

A Bruker Vertex 70 instrument (Billerica, MA, USA) was used to acquire the infrared absorption spectra of pullulan, oxidized pullulan, and the prepared AgNPs. The spectrometer used a scan range from 4000 cm^−1^ to 650 cm^−1^ at a resolution of 2 cm^−1^ and 32 scans. The morphologies of the nanoparticles were determined using a scanning electron microscope type Quanta 200 (FEI Company, Hillsboro, OR, USA) operating in a low vacuum mode at 20 kV. The apparatus has an LFD detector (FEI Company, Hillsboro, OR, USA) and is equipped with an energy dispersive spectroscopy (EDX) module for elemental analysis. 

### 2.6. Antifungal Evaluation

The antifungal activity of the AgNPs on the various pathogenic fungi was assessed using the guidelines of EUCAST E.Def. 7.3.2 for yeasts [39]. The tests were done in an RPMI-1640 medium buffered with MOPS, containing an additional 2% glucose. Firstly, a stock solution with a concentration 200-fold higher than the highest final concentration to be tested was prepared from each compound by dissolving an appropriate amount of the sample in DMSO. The solutions to be tested were prepared by using a serial two-fold dilution starting with the stock solutions, and the following final concentrations were reached: 40, 20, 16, 12, 8, 4, 2, 1, 0.5, and 0.25 mM (as Ag final concentrations). The 96-well microplates incorporating the serial dilutions of the analyzed samples were prepared and stored in a freezer at −20 °C before use. The final content of the inoculum was regulated to 10^5^ CFU/mL. The minimum inhibitory concentration (MIC) threshold was spectrophotometrically determined, measuring the absorbance at 450 nm via an MR-96A microplate reader after 24 or 48 h (depending on the species) of incubation at 36 °C. The MIC endpoint is determined as the lowest concentration of the analyzed sample for which a decrease of at least 50% in growth, compared to that of the positive control (no tested compound in the well), was observed, considering the optical density measurements. *C. parapsilosis* ATCC 22019 and fluconazole were used for the quality control of each test. Both pullulan and oxidized pullulan were tested for their antifungal activity using the same methodology as above. The range of tested concentrations was identical to that of AgNPs. 

## 3. Results

### 3.1. Characterization of Oxidized Pullulan

**Figure 1:**
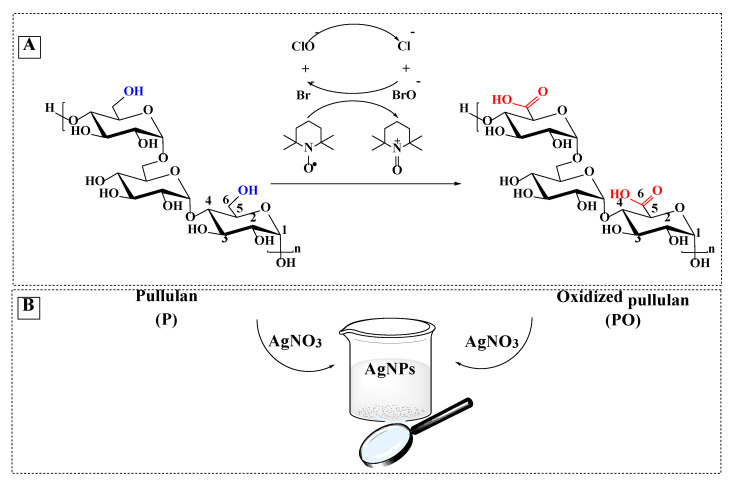

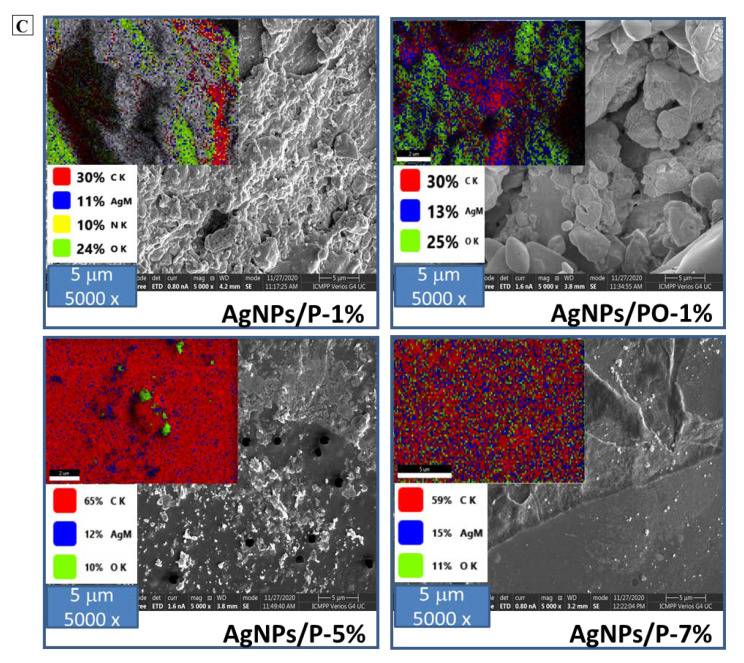
Selective oxidation of C6 primary OH groups of pullulan to carboxylate moieties by TEMPO/sodium hypochlorite/sodium bromide system, in water at pH 10 (**A**); simplified scheme of the silver nanoparticles generation in the presence of pullulan/ oxidized pullulan (**B**) and SEM images of silver nanoparticles prepared by using different concentration of pullulan or oxidized pullulan and AgNO_3_ (**C**).

Pullulan was oxidized at room temperature for 2 h, by using TEMPO as a mediator for the oxidation reaction and sodium hypochlorite/sodium bromide to convert the primary OH groups to carboxylic groups (Figure 1A). TEMPO is a nitroxyl radical with an unpaired electron delocalized between the N and O atoms, which during the oxidation process is converted into a nitrosonium ion—the actual oxidant—under alkaline conditions (pH = 10). The pH decreases continuously during the oxidation process, and it is, therefore, necessary to maintain the pH around 10 and thus maintain the stability of the reaction. A stable pH ensures the high quality of products. The dissociation of the primary hydroxyl groups is favored under an alkaline medium so that the appearance of the covalent bonds occurs faster than in neutral or acidic environments.

**Figure 2:**
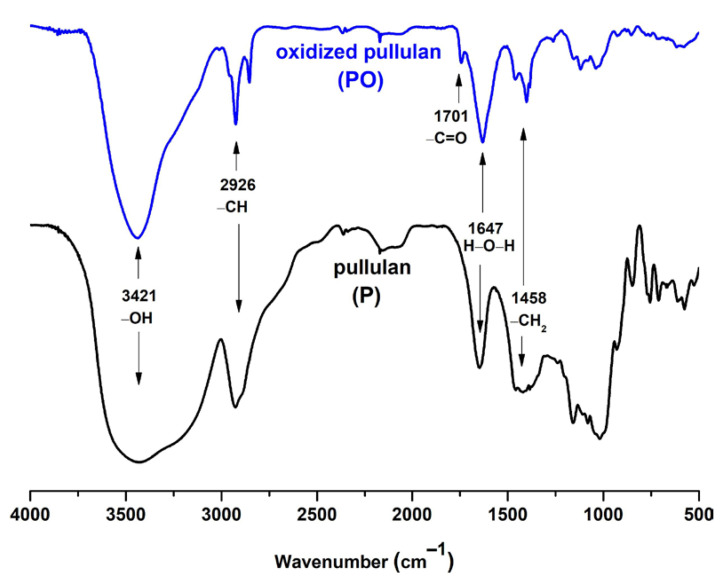
FTIR spectra of pullulan (**bottom**) and its TEMPO-mediated oxidation product (**top**).

The oxidation of pullulan can be observed easily in FTIR (Figure 2) or the NMR (Figure S1, Supporting Information). To give rise to the CO vibration band specific to the carboxylic groups, the oxidized pullulan was acidified using a diluted HCl solution. Following the acidification process, the carboxylate groups were transformed into their acidic form. The spectra of the pullulan and oxidized pullulan are the same; the only modification was observed around 1700 cm^−1^ due to the carboxyl groups formed during deoxidation. The typical FTIR bands of pullulan are those characteristic to the OH stretching vibration, between 3100–3600 cm^−1^; C-H stretching vibration, at 2926 cm^−1^; adsorption band of bounded water, located at 1647 cm^−1^; symmetric CH_2_ bending vibration, assigned at 1458 cm^−1^; and C-O-C stretching of α-(1,4)-glycosidic linkages, at 930 cm^−1,^ which can be seen as a marker sign of the “amorphous” region [40]. The adsorption band at 1610 cm^−1^ is assigned to the C=O stretching of the free carboxylate groups; whereas, the band at 1417 cm^−1^ is characteristic of the C-O symmetric stretching of carboxyl groups in their dissociated form [41].

### 3.2. Characterization of AgNPs

Pullulan (P) and oxidized pullulan (PO) have been proposed as reducing agents and stabilizers for the synthesis of silver nanoparticles [36]. The pullulan or oxidized pullulan contains abundant hydroxyl and carboxylic groups, respectively, which facilitate the complexation of Ag^+^ to the polymer matrix and the reduction of the Ag^+^ to the Ag^0^. Four experiments were conducted using pullulan or oxidized pullulan, the concentration of silver nitrate and polymer being variable (Table 1). 

Consisting of multiple hydroxyl groups, pullulan confers a steric stabilization, the hydrogen bonds being capable of coordinating Ag, providing stability for a longer time. On the other hand, oxidized pullulan confers electrosteric stabilization, the COOH groups leading to the appearance of some electrostatic repulsion between the negatively charged core–shell nanoparticles [36]. 

**Figure 3:**
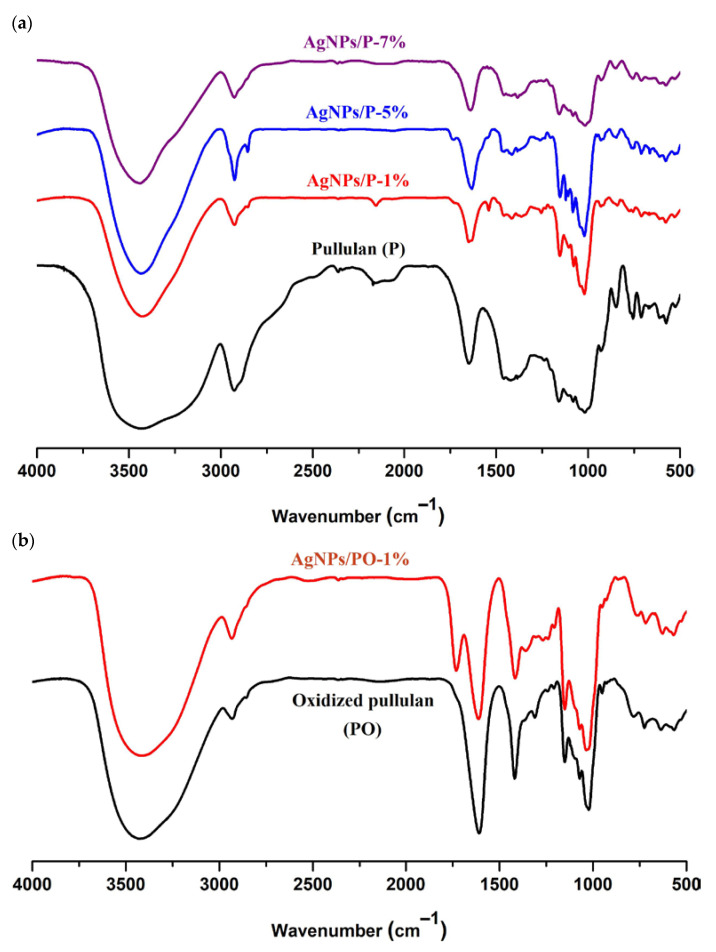
FTIR spectra of pullulan (P) and silver nanoparticles stabilized by P (**a**), and oxidized pullulan (PO) and silver nanoparticles stabilized by PO samples (**b**).

The FTIR spectra of the AgNPs stabilized by pullulan/oxidized pullulan are shown in Figure 3. The FTIR spectra of the nanoparticles differ slightly from the spectra of pullulan: the band at 3421 cm^−1^ corresponding to the pullulan is shifted to 3435 cm^−1^ in the case of pullulan-silver nanoparticles, and the band from 1647 cm^−1^ is shifted slightly to lower wavenumbers. Moreover, the band from 2926 cm^−1^ (corresponding to the C-H bond) is shifted to 2924 cm^−1^. These observations clearly show the strong interaction between silver and the hydroxyl groups in pullulan (Figure 3a). Comparing the FTIR spectrum of oxidized pullulan with the FTIR spectrum of PO-mediated silver nanoparticles (AgNPs/PO-1%), we noticed that the IR absorption intensities for ν_s_ C=O (1418 cm^−1^) and ν_as_ C=O (1608 cm^−1^) of deprotonated carboxylic acid groups are visibly decreased. These findings support the hypothesis of the binding of carboxylic groups with Ag nanoparticles through the free carboxylate group (Figure 3b). In the spectra of oxidized pullulan nanoparticles (AgNPs/PO-1%), a new adsorption band as a shoulder, around 1736 cm^−1^, appears due to carboxylate groups involved in the attachment of silver atoms. Based on this evidence, it can be inferred that both the hydroxyl (in pullulan and oxidized pullulan) and carboxyl (exclusively on oxidized pullulan) groups are responsible for the stabilization of silver nanoparticles [42,43].

**Figure 4:**
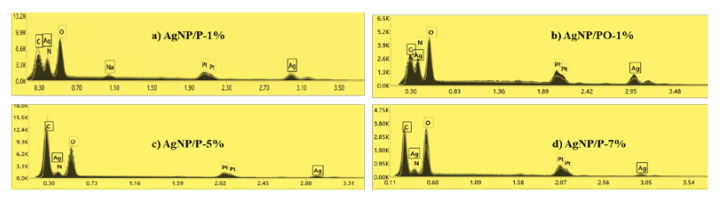
EDX spectra of synthesized silver nanoparticles: (**a**) AgNP/P-1%, (**b**) AgNP/PO-1%, (**c**) AgNP/P-5%, and (**d**) AgNP/P-7% observed at 3 keV binding energy for silver.

Figure 1C shows SEM micrographs of the samples AgNPs/P-1%, AgNPs/PO-1%, AgNPs/P-5%, and AgNPs/P-7%. From images, it is apparent that the AgNPs have many irregular shapes of different sizes. Additionally, the presence of the AgNPs clusters is observed, which may be due to the aggregation of nanoparticles formed during sample preparation. Figure 4 highlights the presence of elemental silver in the EDX spectra of the AgNPs samples, confirmed by the presence of a peak at ~3 keV.

### 3.3. Antifungal Activity

**Figure 5:**
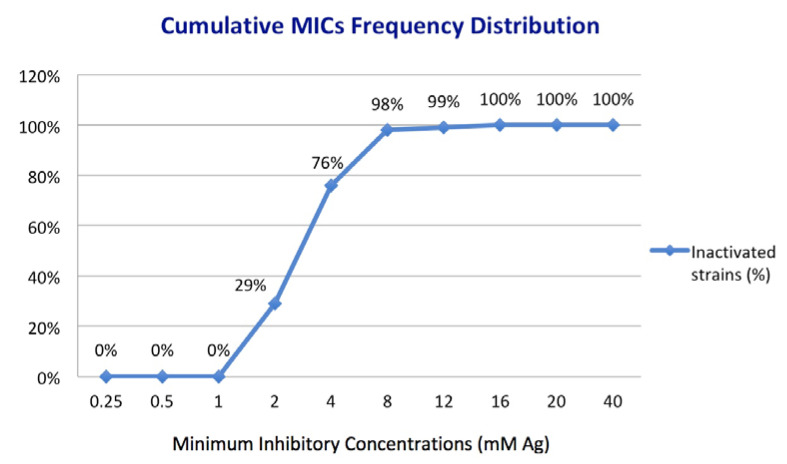
Cumulative distribution of MICs frequency for 100 clinical isolates based on the concentration of Ag as an active element of AgNPs.

All four types of silver nanoparticles exhibited antifungal activity against the clinical isolates within the limits of the tested concentrations (Figure 5). It is important to note that the MIC of each AgNPs (expressed as mM Ag) was the same for a given isolate, meaning a similar release pattern of Ag. Thus, the tested compounds inhibited 29% of the isolates at 2 mM Ag, 76% at 4 mM Ag, 98% at 8 mM Ag, and 99% at 12 mM Ag. AgNPs successfully inhibited all tested isolates at concentrations higher than 16 mM Ag. The most resilient fungal isolates belong to *C. parapsilosis* (one isolate with MIC: 12 mM Ag) and *C. famata* (one isolate with MIC: 16 mM Ag). Among the isolates of the most represented species in the study (i.e., *C. glabrata* n = 27; *C. albicans* n = 23; and *C. parapsilosis* n = 16), there is no particular MIC for the tested AgNPs, the range of active concentrations varying from 2 to 12 mM with a predominance of 4 and 8 mM values.

## 4. Discussion

There is plenty of evidence about the antifungal activity of silver nanoparticles. For example, silver nanoparticles between 3 and 15 nm are very active against different phytopathogens (part of the genera Alternaria, Botrytis, Cladosporium, Cylindrosporium, Phytum, Fusarium et al.). Concentrations of 100 ppm are capable of completely inhibiting their growth [27]. Previous studies demonstrate a correlation between the size of the nanoparticles and their antifungal action. Small nanoparticles appear to be more active against phytopathogenic fungi; for example, silver nanoparticles with a size of 3 nm at a concentration of 50 ppm reduce the growth of Fusarium oxysporum f. sp. radicis-lycopersici (FORL cu 90%) [44].

Different types of silver nanoparticles are known to have antifungal activity against the clinical isolates of human pathogens, for example, against *Candida spp*. and dermatophytes [45]. Such activity is manifested by both engineering nanoparticles and Bio-AgNPs. For example, chitosan-conjugated Bio-AgNPs at concentrations of 50 mg/L have an antifungal effect against most clinical *Candida* isolates tested. The morphological study demonstrates the significant, harmful effects that pathogen cells suffer under the action of these nanoparticles [46]. Another type of silver nanoparticles obtained by biosynthesis and stabilized with starch tested on the *Candida albicans* BWP17 isolate was proven to have significant antifungal activity, with a minimum inhibitory concentration (at 80% inhibition, MIC80) of 280 µg/mL [47]. Silver nanoparticles stabilized in hydrolyzed collagen and natural polymers (carrageenan, agar) demonstrated the high inhibitory capacity of the *Candida albicans* wild-type strain SC 5314 at concentrations of Ag varying from 3.9 to 15.62 μM [48]. 

Silver nanoparticles coated with polyvinylpyrrolidone have shown antifungal activity against some dermatophytes, for example, against *Trichophyton rubrum* and *Trichophyton mentagrophytes*, the growth of which is inhibited by 75%, at 13.5 µg/mL nanoparticles concentration [49].

Thus, there is a trend to perform antifungal tests for different types of silver nanoparticles obtained either by engineering or biological synthesis. The effects observed by different authors depend on the size of the nanoparticles, the stabilizer used, the tested concentrations, and on the species of fungi that was assessed. Works reflecting the results obtained from clinical isolates of fungi are infrequent. In such contexts, our findings are even more valuable by demonstrating the efficiency of four types of silver nanoparticles against 19 species of pathogenic fungi. Our study demonstrates that silver nanoparticles exhibit uniform antifungal activity against all tested fungal isolates at relatively close concentrations. For the vast majority of the 100 isolates tested (98 out of 100), the concentration of nanoparticles that completely inhibit fungal growth is up to 8mM Ag. This fact reveals these nanomaterials as alternative antifungal remedies or adjuvants to existing therapies, especially in relation to infections with isolates resistant to current antifungal medication. Concerning their mechanism of action, we can hypothesize that nanoparticles induce serious impairments of the cell membrane and cell wall and serious damages and degeneration of the organelles of yeast cells, resulting in a severe perturbation of metabolism and death. Xia et al. (2016) have previously described these findings after performing transmission electron microscopy on the yeast species *Trichosporon asahii* cells pre-treated with AgNPs [50].

Further studies are required to fully expound on the action mechanism and the pharmacokinetics of the silver nanoparticles after their administration to live organisms.

## 5. Conclusions

Pullulan, both native and its 6-carboxy counterpart, has proven to be an efficient matrix for reducing and stabilizing silver nanoparticles. This study has demonstrated the high antifungal activity of silver nanoparticles in vitro but requires further investigation in vivo, and proper standardization, stabilization, and toxicology to make them applicable as antimicrobials/antifungals. The presented data demonstrate that silver nanoparticles have relatively uniform antifungal activity against all tested fungal isolates at relatively close concentrations.

## Figures and Tables

**Table 1:** Sample codes for the silver nanoparticles synthesized using pullulan (P) and oxidized pullulan (PO) as reducing/stabilizing agents.

Code	Reducing/Stabilizing Polymer	AgNO_3_ Conc. (%)
AgNPs/P-1%	Pullulan 1%	4.45
AgNPs/PO-1%	Oxidized pullulan 1%	4.45
AgNPs/P-5%	Pullulan 5%	0.64
AgNPs/P-7%	Pullulan 7%	1.91

## Data Availability

The data presented in this study are available on request from the corresponding author.

## Supplementary Materials

The following are available online at https://www.mdpi.com/article/10.3390/ma14227041/s1, Figure S1: ^13^C-NMR spectra of the pullulan before oxidation (A) and the pullulan obtained after oxidation in the presence of the TEMPO/NaClO/NaBr (B)., Figure S2: The UV-Vis spectra of the silver nanoparticles (pullulan: AgNPs/P-1% and oxidized pullulan: AgNPs/PO-1%) during synthesis, at room temperature.
