# Supplementary material for: Synthesis, Characterization, and Antifungal Activity of Silver Nanoparticles Embedded in Pullulan Matrices
Source: Materials (Basel). 2021 Nov 20;14(22):7041. doi: 10.3390/ma14227041 (PMC8622049; doi:10.3390/ma14227041)
Supplement: Supplementary file 1 [file materials-14-07041-s001.zip › materials-1455673-supplementary.pdf]

materials

Article

Synthesis, Characterization, and Antifungal Activity of Silver Nanoparticles Embedded in Pullulan Matrices

Olga Burduniuc [1,2,*], Andra-Cristina Bostanaru [3], Mihai Mares [3,*], Gabriela Biliuta [4,*] and Sergiu Coseri [4,*]

[1] "Nicolae Testemitanu" State University of Medicine and Pharmacy, 165, Stefan cel Mare blvd., MD 2001 Chisinau, Moldova
[2] National Public Health Agency, 67A Gheorghe Asachi, MD 2028 Chisinau, Moldova
[3] Laboratory of Antimicrobial Chemotherapy, Faculty of Veterinary Medicine, "Ion Ionescu de la Brad" University of Life Sciences (IULS), 8 Mihail Sadoveanu Alley, 700489 Iasi, Romania; acbostanaru@uaiasi.ro
[4] "Petru Poni" Institute of Macromolecular Chemistry, 41A Grigore Ghica Voda Alley, 700487 Iasi, Romania
* Correspondence: olga.burduniuc@ansp.gov.md (O.B.); mycomedica@gmail.com (M.M.); biliuta.gabriela@icmpp.ro (G.B.); coseris@icmpp.ro (S.C.)

Citation: Burduniuc, O.; Bostanaru, A.-C.; Mares, M.; Biliuta, G.; Coseri, S. Synthesis, Characterization, and Antifungal Activity of Silver Nanoparticles Embedded in Pullulan Matrices. *Materials* **2021**, *14*, 7041. https://doi.org/10.3390/ma14227041

Academic Editor: Anna Karewicz

Received: 25 October 2021
Accepted: 16 November 2021
Published: 20 November 2021

Publisher's Note: MDPI stays neutral with regard to jurisdictional claims in published maps and institutional affiliations.

Copyright: © 2021 by the authors. Licensee MDPI, Basel, Switzerland. This article is an open access article distributed under the terms and conditions of the Creative Commons Attribution (CC BY) license (http://creativecommons.org/licenses/by/4.0/).

Nuclear magnetic resonance spectroscopy

Nuclear Magnetic Resonance (NMR) Spectroscopy spectra were realized with a Bruker-Avance DR X 400 MHz Spectrometer, equipped with a 5 mm QNP direct detection probe and z-gradients. Solutions in D_2O of pullulan and oxidized pullulan using tetramethylsilane TMS ($\delta = 0$ ppm) as internal standard were used to record the ^{13}C-NMR spectra.

The oxidation is clearly seen by ^{13}C-NMR. The presence of the C signal at 175 ppm, characteristic for the −COOH groups, reveal that the oxidation process at primary −OH groups was occurred.

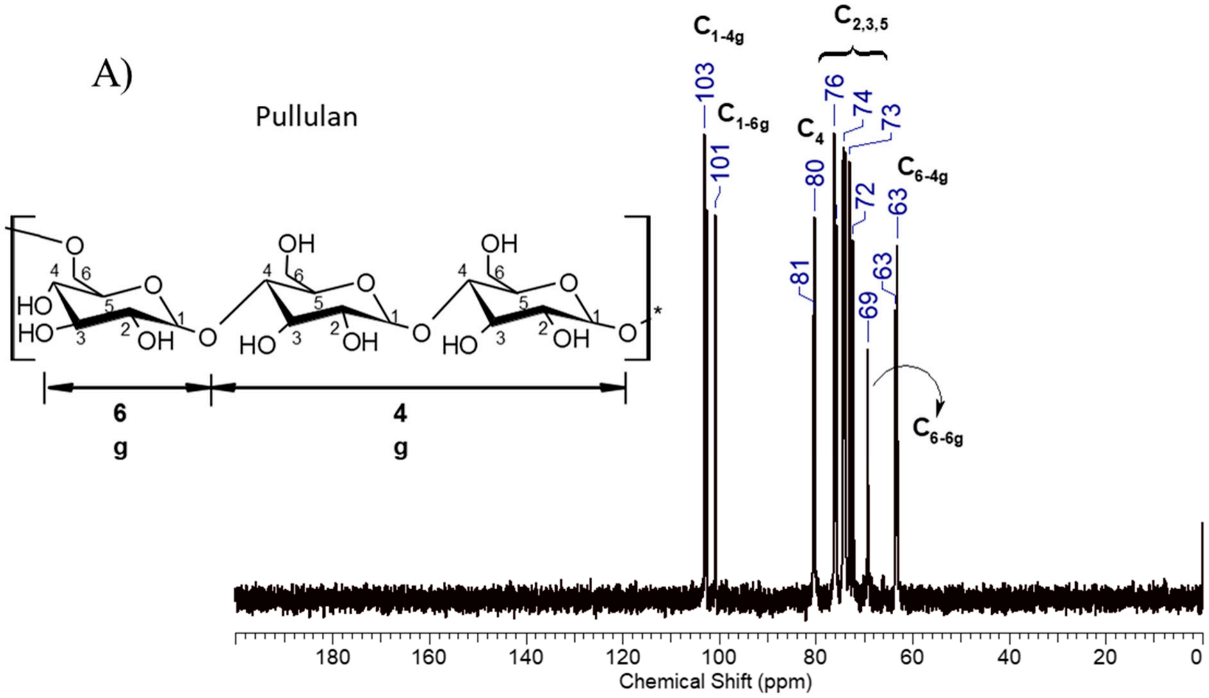

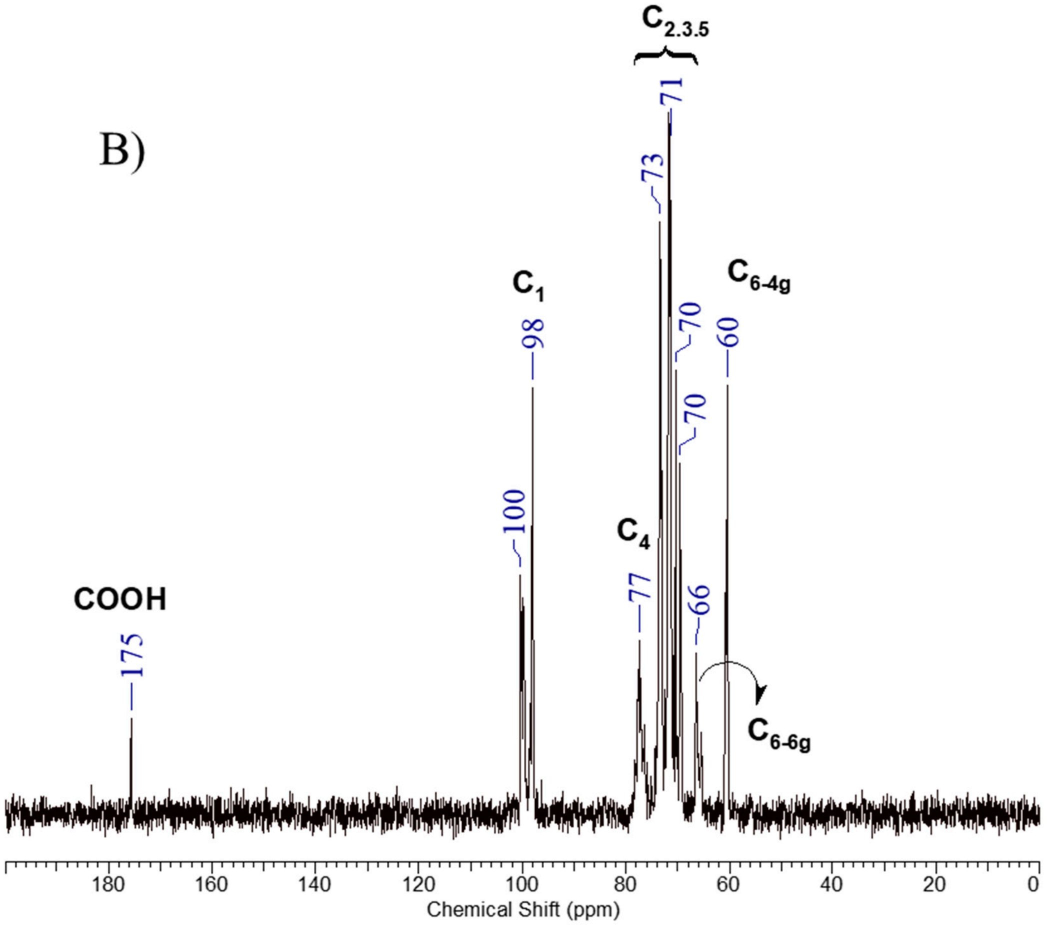

Figure S1. ^{13}C-NMR spectra of the pullulan before oxidation (A) and the pullulan obtained after oxidation in the presence of the TEMPO/NaClO/NaBr (B).

UV-visible spectrophotometer measurements

The AgNPs prepared by using pullulan or oxidized pullulan were characterized by using ultraviolet-visible (UV-vis) spectroscopy. The optical absorption properties of AgNPs samples were measured using a SPECORD 200 Analytik Jena spectrometer over the range of 300 – 700 nm. The effect of the reducing/stabilizing agent on the AgNPs formation is illustrated by recording the UV-Vis spectra, **Figure S2**. It is well known that silver nanoparticles absorb radiation in the visible region of the electromagnetic spectrum (380-450 nm) due to the SPR transition.

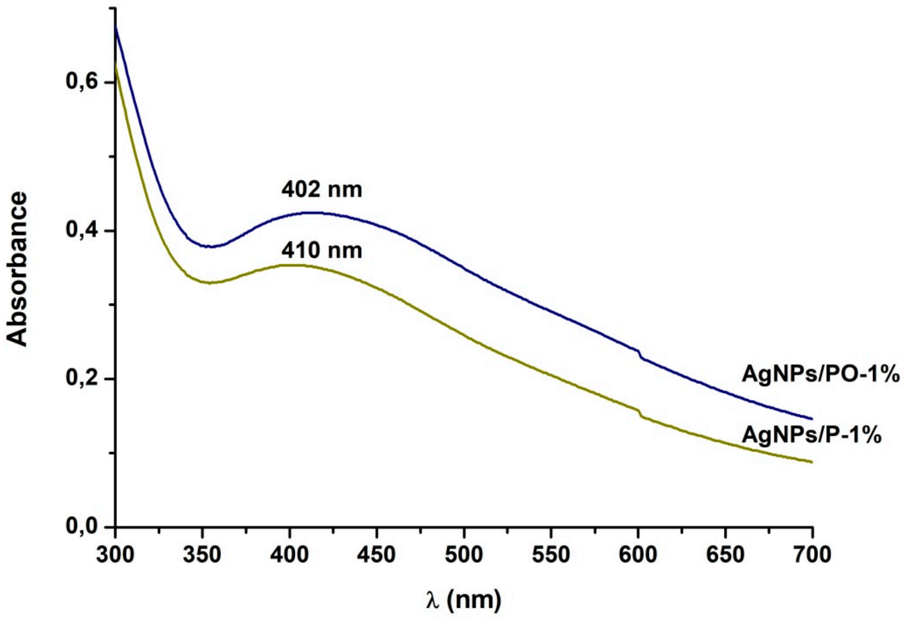

Figure S2. The UV-Vis spectra of the silver nanoparticles (pullulan: AgNPs/P-1% and oxidized pullulan: AgNPs/PO-1%) during synthesis, at room temperature.
